# Efficient Synthesis of Furfural from Biomass Using SnCl_4_ as Catalyst in Ionic Liquid

**DOI:** 10.3390/molecules24030594

**Published:** 2019-02-07

**Authors:** Yifan Nie, Qidong Hou, Weizun Li, Chuanyunlong Bai, Xinyu Bai, Meiting Ju

**Affiliations:** Tianjin Engineering Research Center of Biomass Solid Waste Resources Technology, College of Environmental Science and Engineering, Nankai University, Tianjin 300071, China; 2120170635@mail.nankai.edu.cn (Y.N.); liweizun@nankai.edu.cn (W.L.); 1510958@mail.nankai.edu.cn (C.B.); 1510958@mail.nankai.edu.cn (X.B.)

**Keywords:** xylose, lignocellulose, furfural, dehydration, ionic liquid

## Abstract

Furfural is a versatile platform molecule for the synthesis of various chemicals and fuels, and it can be produced by acid-catalyzed dehydration of xylose derived from renewable biomass resources. A series of metal salts and ionic liquids were investigated to obtain the best combination of catalyst and solvent for the conversion of xylose into furfural. A furfural yield of 71.1% was obtained at high xylose loading (20 wt%) from the single-phasic reaction system whereby SnCl_4_ was used as catalyst and ionic liquid 1-ethyl-3-methylimidazolium bromide (EMIMBr) was used as reaction medium. Moreover, the combined catalyst consisting of 5 mol% SnCl_4_ and 5 mol% MgCl_2_ also produced a high furfural yield (68.8%), which was comparable to the furfural yield obtained with 10 mol% SnCl_4_. The water–organic solvent biphasic systems could improve the furfural yield compared with the single aqueous phase. Although these organic solvents could form biphasic systems with ionic liquid EMIMBr, the furfural yield decreased remarkably compared with the single EMIMBr phase. Besides, the EMIMBr/SnCl_4_ system with appropriate water was also efficient to convert xylan and lignocellulosic biomass corn stalk into furfural, obtaining furfural yields as high as 57.3% and 54.5%, respectively.

## 1. Introduction

With the depletion of fossil energy and the deterioration of ecological environments, modern society needs to develop economical, energy-efficient, and environmentally friendly processes to achieve sustainable production of fuels and chemicals [1]. In recent years, considerable attention has been paid to utilizing renewable biomass resources to produce value-added chemicals to relieve the resource and energy crisis [2,3,4]. Both chemical catalysis and biotechnology have been proposed to depolymerize cellulose and hemicellulose into C_5_–C_6_ sugar monomers, such as glucose, xylose, and arabinose [5,6,7]. C_6_ and C_5_ sugar can be further transformed into 5-hydroxymethylfurfural (HMF) and furfural by chemical catalysis routes, respectively [8,9]. Both HMF and furfural could be used as starting materials for the synthesis of important furan derivatives. However, the commercial application of HMF is still not realized due to the high cost of HMF production [10,11,12,13]. In contrast, the large-scale production and commercial application of furfural have already been achieved. Moreover, the important furfural derivatives, such as furfural alcohol, tetrahydrofurfuryl alcohol, furan, tetrahydrofuran (THF), dihydropyran, acetylfuran, furanamine, and furan acid have showed great potential in industry [14,15,16,17]. Among these chemicals, furfural alcohol accounts for 62% of the global furfural market. Furfural can also be converted into green fuels such as methyl furan, methyl tetrahydrofuran, valerate, ethyl furan, and ethyltetrahydro furan ethers [18,19]. 

Currently, furfural is industrially produced from pentosan-rich biomass via pentose cyclodehydration by using mineral acids (H_2_SO_4_ or HCl) as catalyst at high temperature [14]. In this process, C_5_ polysaccharides (primarily xylan) are first hydrolyzed into monosaccharides (primarily xylose) by acid and then the formed monosaccharides are dehydrated to furfural. Subsequently, furfural product is usually recovered from the liquid phase by steam stripping to avoid further degradation and then purified by double distillation [20,21]. However, the commercial furfural production process suffers from a number of drawbacks, including relative low furfural yield (around 45–55%), high energy consumption, equipment corrosion, impractical catalyst recovery, as well as environmental hazards [13]. Therefore, it is very important to develop efficient, inexpensive, and environmentally sustainable furfural production process. 

The conversion of xylose into furfural usually involves the use of either homogeneous acids [22,23,24,25,26] or heterogeneous solid acids [27,28,29,30,31] as catalysts in single-phasic [22,24,32] or biphasic reaction systems [27,33]. Some metal chlorides with Lewis acidity (CrCl_3_, AlCl_3_, and FeCl_3_) exhibit high catalytic activity in the water–organic solvent biphasic systems [23,31,34]. In these biphasic systems, the produced furfural is extracted into the organic phase instantaneously, which inhibits the side reaction of furfural and increases the final yield of furfural significantly [35,36,37]. However, the realistic conversion efficiency is quite low in view of the low loading of sugar substrate and harsh reaction conditions [38,39]. Moreover, these biphasic systems require a large amount of organic solvents and electrolytes (such as NaCl) to improve the extraction efficiency, which leads to the increase of cost and concomitant pollutions [40].

Ionic liquids show many advantages over water and organic solvent systems, including very low volatility, good dissolving capacity, chemical and thermal stability, and improved reaction efficiency [19]. Zhang et al. investigated the conversion of xylan into furfural in BMIMCl under microwave irradiation by using metal chlorides as catalysts; AlCl_3_ resulted in the highest furfural yield of 84.8% at 170 °C for 10 s [24]. However, the high furfural yield, to a large extent, was attributed to the low substrate loading (the xylose loading was 1.9 wt%, relative to the mass of solvent) and microwave heating. Peleteiro et al. investigated the conversion of xylose to furfural in ionic liquid (BMIMCl) in the presence of CrCl_3_ with a relative high substrate loading of 10 wt%, obtaining a furfural yield of 50% operating at 120 °C for 30 min [41]. Although the furfural yield was not as high as that in the biphasic system, this work indicated that the xylose conversion efficiency can be remarkably improved by the use of ionic liquid. Furthermore, ionic liquids with strong Bronsted acidity (acidic ionic liquids) can perform as both solvents and catalysts, enabling the direct conversion of sugars into furans [42,43]. The acidic ionic liquids BMIMHSO_4_ and BMIMMeSO_3_ have been employed to produce furfural from Miscanthus, obtaining furfural yields of 33% and 13%, respectively (120 °C, 22 h) [44]. 

In the present work, furfural was produced from xylose, xylan, and corn stalk in the medium of ionic liquid. A series of metal salts and ionic liquids were investigated to obtain the best combination of catalyst and solvent. The effects of reaction condition, catalyst dosage, and substrate loading on the yield of furfural were investigated to improve the furfural yield. Besides, the ionic liquid organic solvent biphasic systems were also tested for the production of furfural from xylose. 

## 2. Results and Discussion

### 2.1. Effect of Catalysts on the Dehydration of Xylose into Furfural

**Figure 1:**
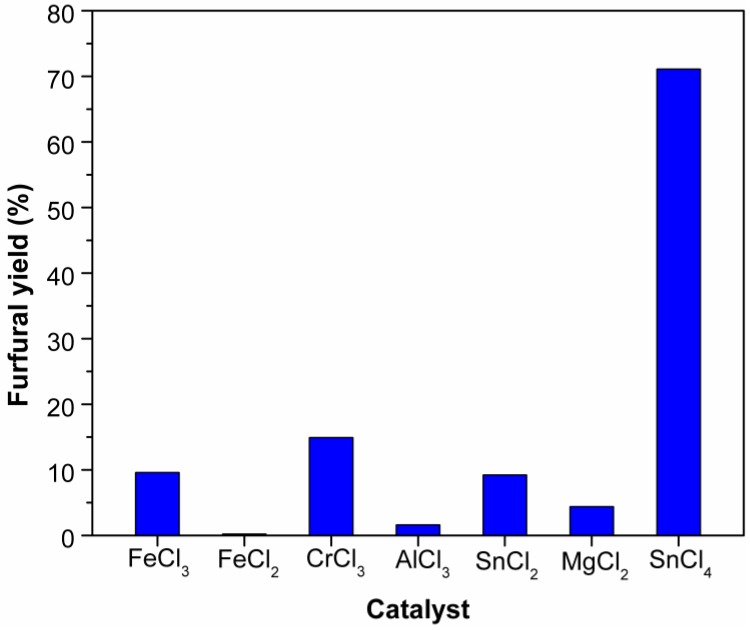
Effect of catalysts on the dehydration of xylose into furfural in EMIMBr. Reaction conditions: 200 mg of xylose was dissolved in 1000 mg of EMIMBr; molar ratio of catalyst:xylose = 1:10; 130 °C; 1 h.

Metal chlorides (FeCl_3_, FeCl_2_, CrCl_3_, AlCl_3_, SnCl_2_, SnCl_4_, MgCl_2_) were compared as catalyst for converting xylose into furfural in ionic liquid EMIMBr under the same reaction conditions. As shown in Figure 1, among these metal chlorides, SnCl_4_ exhibited better catalytic performance than other tested catalysts, obtaining a furfural yield as high as 71.1%. In contrast, the furfural yields over CrCl_3_, FeCl_3_, and SnCl_2_ were 14.9, 9.6, and 9.2%, respectively. As for AlCl_3_, FeCl_2_, and MgCl_2_, the furfural yields were less than 5%. It was observed that the reaction mixture became brown quickly in the thick-walled glass vials, suggesting the formation of humins. The extremely low yields from CrCl_3_, AlCl_3_, and FeCl_3_ can be explained by the aggravation of strong side reactions. In addition, the catalytic effect of metal sulfates, Fe_2_(SO_4_)_3_ and FeSO_4_, for the conversion of xylose into furfural were also investigated. However, almost no furfural was detected when using these metal sulfates as catalyst, suggesting that these two metal sulfates had no catalytic effect in the medium of EMIMBr.

**Figure 2:**
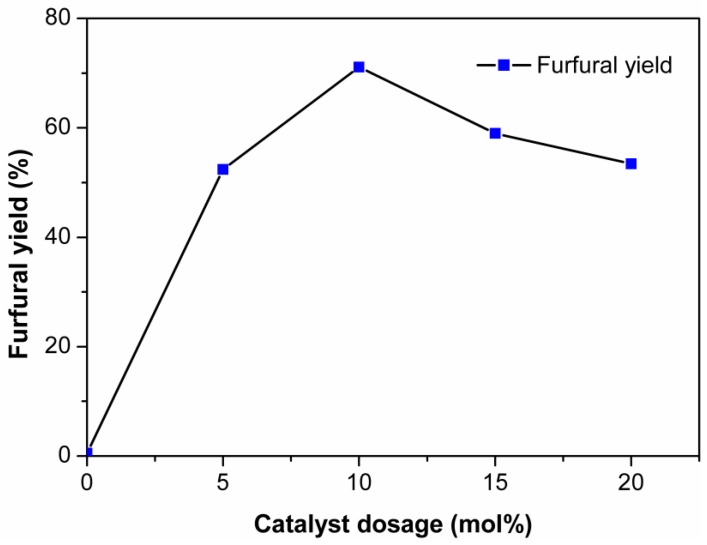
Effect of catalyst dosage on the dehydration of xylose into furfural. Reaction conditions: 200 mg of xylose was dissolved in 1000 mg of EMIMBr; 130 °C; 1 h.

The concentration of the acidic species in the reaction medium was determined by the catalyst dosage [45,46]. Consequently, the catalyst dosage had a remarkable effect on the dehydration of xylose into furfural. Figure 2 shows the effect of catalyst dosage on the dehydration of xylose into furfural. When the catalyst dosage increased from 5 mol% to 10 mol%, the furfural yield increased from 52.4% to 71.1%. As the catalyst dosage further increased, the furfural yield gradually decreased. Excessive catalyst not only accelerated the formation of furfural from xylose but also promoted the side reactions, thus leading to the reduced furfural yield [14]. 

**Figure 3:**
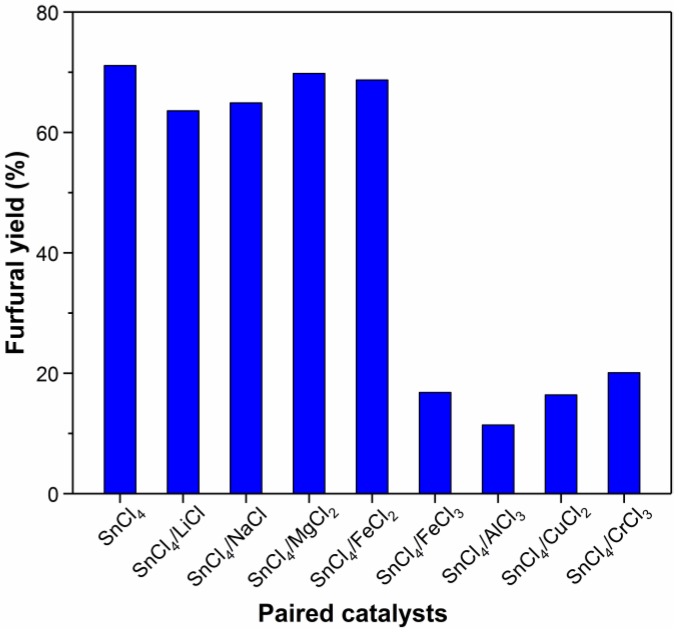
Effect of different paired catalysts on the dehydration of xylose into furfural. Reaction conditions: 200 mg xylose, 1000 mg of EMIMBr; molar ratio of catalyst:xylose = 1:10, SnCl_4_ and another metal chloride molar ratio 4:1; 130 °C; 1 h.

It has been reported that the combined use of two metal chlorides may improve furfural yield in 1-ethyl-3-methyl-imidazolium chloride (EMIMCl) or a water–organic solvent system [24,47]. For example, Zhang et al. found that the furfural yield from corncob catalyzed by combined catalysts CrCl_3_/AlCl_3_ was higher than single CrCl_3_ or single AlCl_3_ [24]. The effect of the combination of SnCl_4_ and another chloride as paired catalysts on the yield of furfural was investigated by Wang et al [47]. They found that the combination of SnCl_4_ with monovalent chlorides, such as LiCl, KCl, and NaCl, could improve the furfural yield. Among these monovalent chlorides, LiCl had the best promotion effect. Moreover, similar results were obtained for the preparation of HMF. Su et al. found that a pair of metal chlorides (CuCl_2_ and CrCl_2_) as catalyst was more conducive for the conversion of cellulose into HMF than the single metal chloride [48]. Therefore, paired catalysts were tested for production of furfural from xylose in ionic liquid EMIMBr. As shown in Figure 3, we chose nine kinds of metal chlorides to pair with SnCl_4_ as catalyst, but the furfural yields from these paired catalysts were all lower than that from SnCl_4_. When FeCl_3_, AlCl_3_, CuCl_2_, and CrCl_3_ were used as co-catalyst, the yield was extremely low (10–20%), and there was a large number of insoluble humins generated during the reaction. In contrast, a furfural yield of more than 60% was obtained by mixing SnCl_4_ and other metal chlorides, which possess weak catalytic activity (MgCl_2_, FeCl_2_) or hardly no catalytic activity (NaCl, LiCl). Although the furfural yield was not improved by the co-catalyst when the total loading of SnCl_4_ and metal chloride was kept at 10 mol%, the furfural yields (69.8% and 68.7%, respectively.) obtained with SnCl_4_/MgCl_2_ and SnCl_4_/FeCl_2_ were almost comparable to that obtained with single SnCl_4_.

Subsequently, we investigated the effect of different dosages of SnCl_4_ and MgCl_2_ on the dehydration of xylose into furfural. As show in Table 1, an extremely low furfural yield of 4.5% was obtained using single MgCl_2_ as catalyst (Entry 6). The furfural yield was 62.0% when 8 mol% SnCl_4_ was used as catalyst. In contrast, when 2 mol% MgCl_2_ was added to the reaction system, the furfural yield increased by 12.6% in comparison with the single 8 mol% SnCl_4_. When 5 mol% MgCl_2_ was combined with 5 mol% SnCl_4_ as catalyst, the furfural yield was still as high as 68.8%, which was 31.2% higher than that obtained with 5 mol% SnCl_4_ and comparable to that obtained with 10 mol% SnCl_4_. These results showed that MgCl_2_ could improve the catalytic activity of EMIMBr/SnCl_4_ when relative low loading of SnCl_4_ was used. Since magnesium salt was cheaper and safer than tin salts, it was more economical to use the SnCl_4_/MgCl_2_ as catalyst instead of single SnCl_4_. Although several studies have reported that the use of paired catalysts can improve the furfural yield, detailed mechanisms involving the coordination of paired catalysts have not been well studied. We observed that fewer humins were produced in the reaction system with paired catalysts consisting of 5 mol% SnCl_4_ and 5 mol% MgCl_2_ than that with single 10 mol% SnCl_4_. The presence of a higher quantity of Sn^4+^ provided abundant Lewis acidity for the isomerization of xylose to xylulose while encouraging the polymerization of furfural with undesired side products [49]. Caes et al. have shown that MgCl_2_ could promote the conversion of sugars into HMF catalyzed by *ortho*-carboxyl-substituted phenylboronic acids [50]. Similarly, García-Sancho et al. found that the presence of CaCl_2_ in a biphasic water–methyl isobutyl ketone (MIBK) system with γ-Al_2_O_3_ as catalyst notably improved the catalytic performance [51]. They proposed that the interaction between Ca^2+^ and glucose molecules favors the formation of α-d-glucopyranose, thus enhancing glucose conversion to HMF. Likewise, it was inferred that the similar interaction between Mg^2+^ and xylose molecules may have promoted the conversion of xylose to furfural, and then MgCl_2_ and SnCl_4_ contributed cooperatively to the dehydration process. 

### 2.2. Effect of Solvents on the Dehydration of Xylose into Furfural

**Figure 4:**
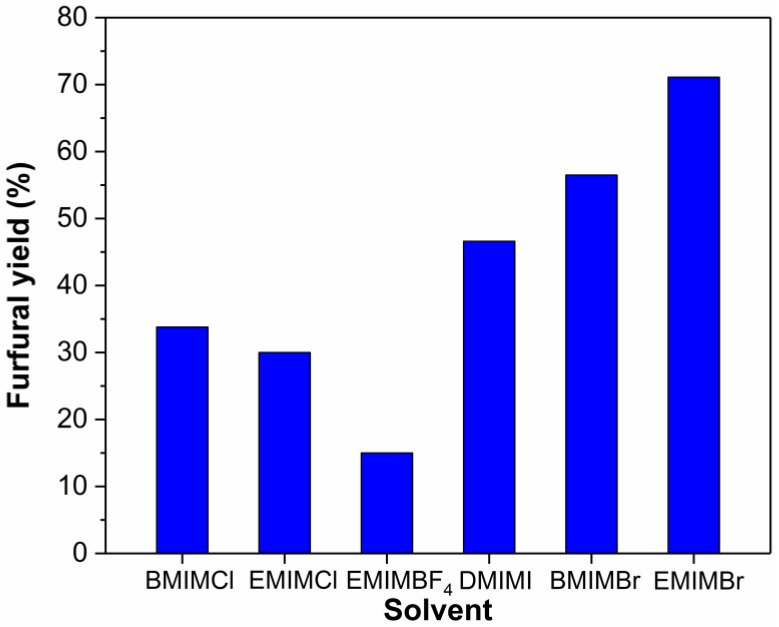
Effect of solvents on the dehydration of xylose into furfural. Reaction conditions: 200 mg of xylose was dissolved in 1000 mg of ionic liquid; molar ratio of SnCl_4_:xylose = 1:10; 130 °C; 1 h.

Figure 4 shows the effect of ionic liquids on the dehydration of xylose into furfural using SnCl_4_. As shown in Figure 4, EMIMBr and 1-butyl-3-methylimidazolium bromide (BMIMBr) resulted in higher furfural yields than other ionic liquids. The furfural yield (56.5%) obtained with BMIMBr was slightly lower than that obtained with EMIMBr. When ionic liquid 1,3-dimethylimidazolium iodide (DMIMI) was used as reaction medium, a furfural yield of 46.7% was obtained. The furfural yields from EMIMCl (30.0%) and BMIMCl (33.8%) were much lower than from EMIMBr and BMIMBr. This observation suggested that Br^–^ containing ionic liquids are more efficient than Cl^−^ containing ionic liquids for the conversion of xylose into furfural catalyzed by SnCl_4_, which may be attributed to the differences in size charge density, and electronegativity of Br^−^ and Cl^−^ [52]. When 1-ethyl-3-methylimidazolium tetrafluoroborate (EMIMBF_4_) was used as reaction medium, the furfural yield was only 15.1%. The low yield from EMIMBF_4_ could be due to their poor ability to dissolve sugar [53]. Besides, the formation of massive brown residues in EMIMBF_4_ indicated the severe occurrence of side reactions.

Furfural yield can be improved by extracting the produced furfural immediately to inhibit its further degradation [33]. The biphasic systems consisting of water and organic solvents, such as THF [54], toluene [9], MIBK [55], and dimethyl carbonate (DMC) [56] have been widely employed for improving both HMF and furfural yield. In addition, the biphasic systems consisting of ionic liquids and organic solvents have also been demonstrated to successfully improve HMF production efficiency [45]. Therefore, in this study, we examined the effect of these organic solvents on the conversion of xylose to furfural in water and EMIMBr. As expected, toluene, DMC, and MIBK could form two phases with water and partially extract the produced furfural from water to the organic phase during the reaction process. For the water–organic solvent biphasic systems, the use of toluene and MIBK as extracting agents could increase the furfural yield noticeably. The furfural yield (45.7%) from the H_2_O–MIBK system was remarkably higher than that obtained from the single water phase with the same sugar loading (20 wt%). In contrast, the furfural yield slightly decreased when DMC was used as extracting agent. In the EMIMBr–organic solvent biphasic systems, THF, toluene, and DMC exhibited high furfural extracting ability from ionic liquid, while ethylene glycol dimethyl ether (EGDE) and MIBK showed poor extracting ability (Table 2). However, the EMIMBr–organic solvent biphasic systems resulted in an obvious decrease of the furfural yield, compared with the single EMIMBr phase system. These results suggested that the adverse effect of organic solvent on the conversion of xylose into furfural in ionic liquid was more predominant than their inhibition to furfural degradation.

According to previous reports, fructose could be converted into HMF (92%) almost quantitatively by EMIMBr without utilizing any other catalyst [49]. To determine the role of EMIMBr in the conversion of xylose to furfural, the xylose conversion in EMIMBr without catalyst was also studied. As shown in Figure 2, almost no furfural was produced when no catalyst was added into the reaction system. This result suggested that EMIMBr could not directly induce the formation of furfural from xylose. Wrigstedt et al. reported that in the preparation of HMF from glucose, bromine ions could promote the fructose dehydration step in the aqueous phase [57]. The isomer of glucose, fructose, was considered as a predominant intermediate in the process of conversion of glucose to HMF. The reaction pathway for conversion of xylose to furfural is analogous to the reaction pathway of the conversion of glucose to HMF [58]. Accordingly, bromine ions probably could also promote the dehydration of the isomer of xylose, xylulose, into furfural. Based on the above analysis, we speculated that bromide anions were able to catalyze the dehydration of ketose (xylulose) to furans, but not the aldose (xylose). In the presence of Lewis acid, xylose was first isomerized into xylulose, and then the formed xylulose was further dehydrated to furfural by EMIMBr.

It is generally accepted that the conversion of xylose catalyzed by Lewis acid involves two steps: the isomerization of xylose into xylulose and the dehydration of xylulose into furfural [22]. As an isomer of xylose, the dehydration of xylulose to furfural is much easier than the direct conversion of xylose to furfural [39]. In the medium of water, little xylulose or fructose was observed since the hydrolysis of SnCl_4_ produced sufficient Bronsted acidity to catalyze the dehydration of the ketose carbohydrates at a rate superior to the isomerization of the aldose carbohydrates [33]. Since the use of ionic liquids could further accelerate the dehydration of ketoses, quantifiable fructose was also not detected in the EMIMBr/SnCl_4_ system [30,45] even though the reduced reaction temperature and higher furfural selectivity suggest that the EMIMBr/SnCl_4_ system prefers the isomerization pathways rather than the direct dehydration pathways.

### 2.3. Effect of Reaction Temperature on the Dehydration of Xylose into Furfural

**Figure 5:**
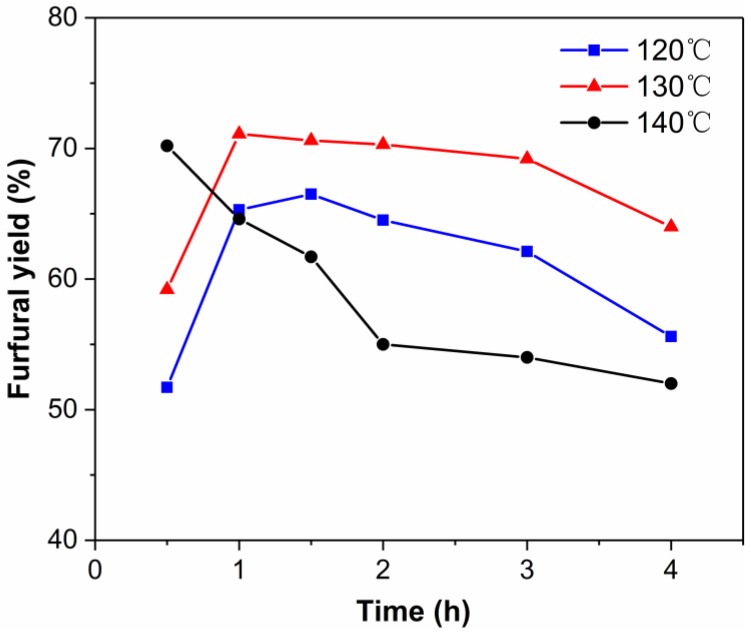
Effect of reaction temperature on the dehydration of xylose into furfural. Reaction conditions: 200 mg of xylose was dissolved in 1000 mg of EMIMBr; molar ratio of SnCl_4_:xylose = 1:10.

**Figure 6:**
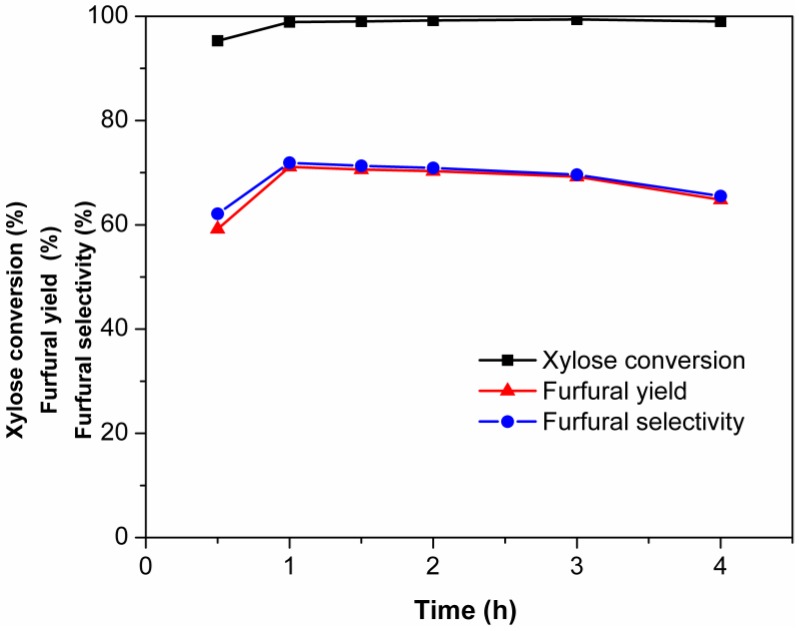
The xylose conversion, furfural yield, and furfural selectivity at 130 °C in the EMIMBr/ SnCl_4_. Reaction conditions: 200 mg of xylose was dissolved in 1000 mg of EMIMBr; molar ratio of SnCl_4_:xylose = 1:10.

Figure 5 shows the effect of reaction temperature on the dehydration of xylose into furfural. It was observed that the rates of xylose conversion and furfural production increased with reaction temperature. The furfural yield first increased and then decreased with the increase of reaction temperature. The maximum furfural yields at 120, 130, and 140 °C were 66.5, 71.1, and 70.2%, obtained at 1.5, 1.0, and 0.5 h, respectively. These results indicated that both the xylose conversion rate and the furfural degradation rate increased with the increasing reaction temperature. This was consistent with previous reports. For example, Wang et al. found that the residence time required to reach the maximum furfural yield decreased with increasing temperature when xylose was converted in water–GVL at 120–150 °C using AlCl_3_ as catalyst [59]. When the temperature was above 130 °C, the degradation was enhanced because of the stronger molecular thermal motion [60,61]. The xylose conversion, furfural yield, and furfural selectivity in the EMIMBr/SnCl_4_ system are shown in Figure 6. The xylose conversion rate reached 95% in 0.5 h. As the reaction time increased from 0.5 h to 1 h, the xylose conversion increased from 95.3% to 98.9% and the furfural selectivity increased from 62.1% to 71.9%. 

### 2.4. Effect of Initial Xylose Loading on the Dehydration of Xylose into Furfural

**Figure 7:**
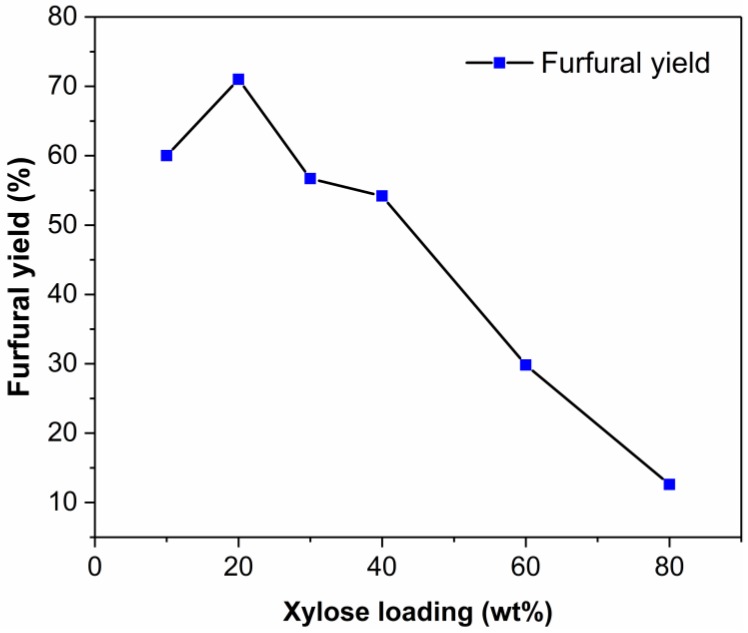
Effect of reaction temperature on the dehydration of xylose into furfural. Reaction conditions: 200 mg of xylose was dissolved in 1000 mg of EMIMBr; molar ratio of SnCl_4_:xylose = 1:10.

The production of furfural from xylose at a high substrate loading is very important for large scale application [62]. Therefore, the effect of initial xylose loading on the dehydration of xylose into furfural was investigated. As shown in Figure 7, furfural yield was markedly affected by the initial xylose loading. When the xylose loading increased from 10 wt% to 20 wt%, the corresponding furfural yield increased from 60.0% to 71.1%. However, the furfural yield decreased with the further increase of xylose loading. A moderate furfural yield of 54.2% was obtained when the xylose loading was 40 wt%. The furfural yield decreased obviously when the initial xylose loading increased from 40 wt% to 80 wt%. When the initial xylose loading was increased to 80 wt%, only 12.6% furfural yield was obtained. It was also observed that the amount of insoluble humins in the reaction bottle increased with the increase of xylose loading. The decrease in furfural yield at high xylose loading may be related to the increase of the viscosity of reaction mixture. The increased viscosity of the mixture may result in unevenly heating in the oil bath reactor [63] and also inhibit the mass transfer between catalyst and reaction substrate [61]. Moreover, the higher xylose concentration may increase the probability of the condensation between furfural and reaction intermediates, leading to the formation of humins [64]. This is consistent with the trend of conversion of xylose in aqueous acidic solutions [63] and ionic liquid EMIMCl [24].

The catalytic performance of the EMIMBr/SnCl_4_ system developed in this work was compared with other representative reaction systems reported recently. As shown in Table 3, unrealistic reaction conditions, including relative high reaction temperature, low xylose loading (lower than 10%), and large amount of organic solvents and electrolytes (such as NaCl) are indispensable to obtain relative high furfural yield from the water–organic solvent biphasic systems [34,65,66]. The furfural yield in the present work was higher than or comparable to previous reports even when the xylose loading (20 wt%, with respect to the mass of reaction medium) was considerably higher than other previous reported catalytic systems. Moreover, the furfural yield still reached 54.2% even at a sugar loading as high as 40 wt%. For the reaction system using SnCl_4_ as catalyst, the furfural yield from EMIMBr was higher than that using other single-phasic reaction media, such as water–DMSO [47] or water [33]. Although the furfural yield up to 69.4% was obtained when using high-pressure CO_2_ as a catalyst in the water/THF system, the xylose loading was only 1.3% [67]. Therefore, EMIMBr/SnCl_4_ is an efficient system for converting carbohydrates to furfural.

### 2.5. Effect of Water Content on the Dehydration of Xylose into Furfural

**Figure 8:**
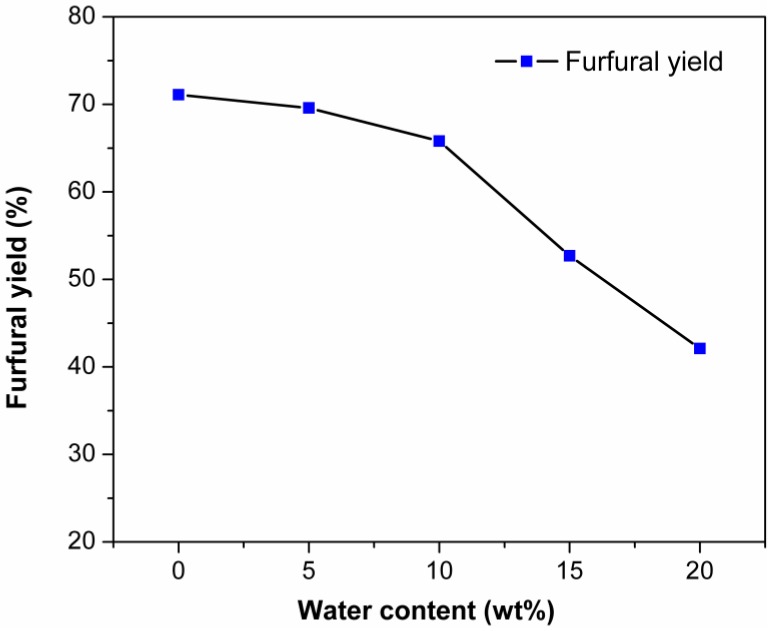
Effect of water content on the dehydration of xylose into furfural. Reaction conditions: 1000 mg of (EMIMBr + H_2_O); molar ratio of SnCl_4_:xylose = 1:10; 130 °C; 1 h.

When xylose is completely dehydrated to furfural, every equivalent of xylose releases three equivalents of water [69]. Previous works indicated that water had a negative effect on the dehydration of xylose into furfural [65,69,70]. The effect of water content on xylose dehydration in EMIMBr/SnCl_4_ system was studied. As shown in Figure 8, the adverse effect of water on the furfural yield was limited when the water content in the system was less than 10 wt%. However, the furfural yield decreased to 52.7% and 42.1% when the water content increased to 15% and 20%, respectively. When ionic liquids were completely replaced with water, the furfural yield was only 22.1%. It was also observed that the amount of insoluble humins also increased with the increase of water content in the system. It was reported that water could lead to undesirable condensation side products and the rehydration of furfural [60,71]. When there was water in the system, the intermediate products would be more active, which may have led to the accumulation of by-products [65,72]. A similar phenomenon was also observed for the dehydration of glucose to HMF in EMIMCl [73].

### 2.6. Conversion of Xylan into Furfural

Xylan, a polymer of xylose, is the predominant component in hemicellulose. In consequence, the conversion of xylan is critical for utilization of realistic biomass feedstocks [22,24]. Zhang et al. proposed that the (AlCl_n_)^(n−3)−^ complexes formed from the BMIMCl/AlCl_3_ system could weaken the glycosidic bonds and then facilitate the hydrolysis of xylan to xylose, which would be further dehydrated into furfural [24]. Likewise, Zhang and Zhao proposed that CrCl_3_ in BMIMCl could weaken the glycosidic bonds through binding with a glycosidic oxygen atom in a similar manner to protic acid, leading to the hydrolysis of xylan to xylose [74]. 

**Figure 9:**
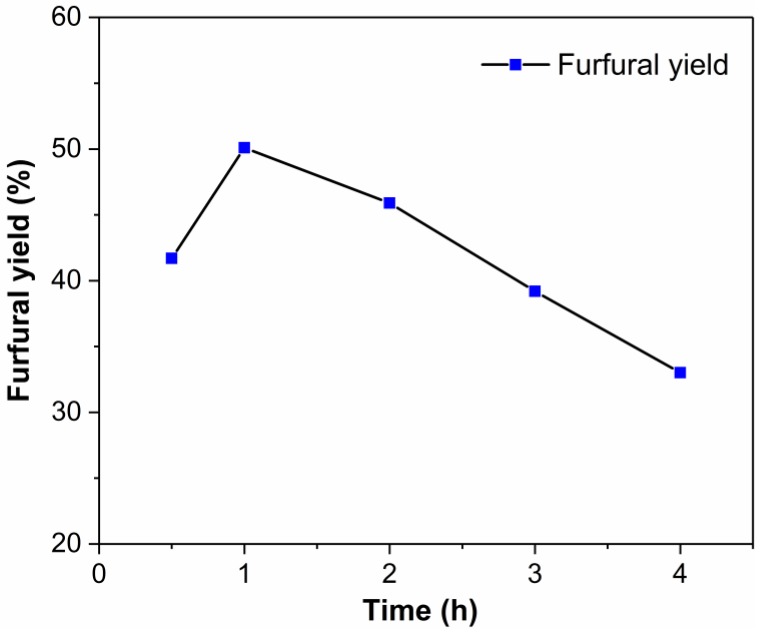
Time profile for the conversion of xylan to furfural in the EMIMBr/SnCl_4_ system. Reaction conditions: xylan 200 mg (1.5 mmol, based on monosaccharide units); 1000 mg of EMIMBr; molar ratio of SnCl_4_:monosaccharide = 1: 10; 130 °C; 1 h.

We attempted to convert xylan into xylose in the EMIMBr/SnCl_4_ system at 130 °C. As shown in Figure 9, the furfural yield was 41.7% after 30 min reaction. The maximum furfural yield of 50.1% was obtained at 1 h. When the reaction time was prolonged to 4 h, the furfural yield declined to 33.0%. Our results showed that the optimized reaction conditions for xylose were also suitable for the conversion of xylan. Binder et al. investigated the conversion of xylan using CrCl_2_ in DMA–LiCl into furfural under identical reaction conditions (at 100 °C) with xylose [22]. However, only trace yields of furfural were obtained, suggesting that these conditions were too mild to accomplish the depolymerization of xylan into xylose. The furfural yield increased to 7–8% when the reaction temperature increased to 140 °C. 

**Figure 10:**
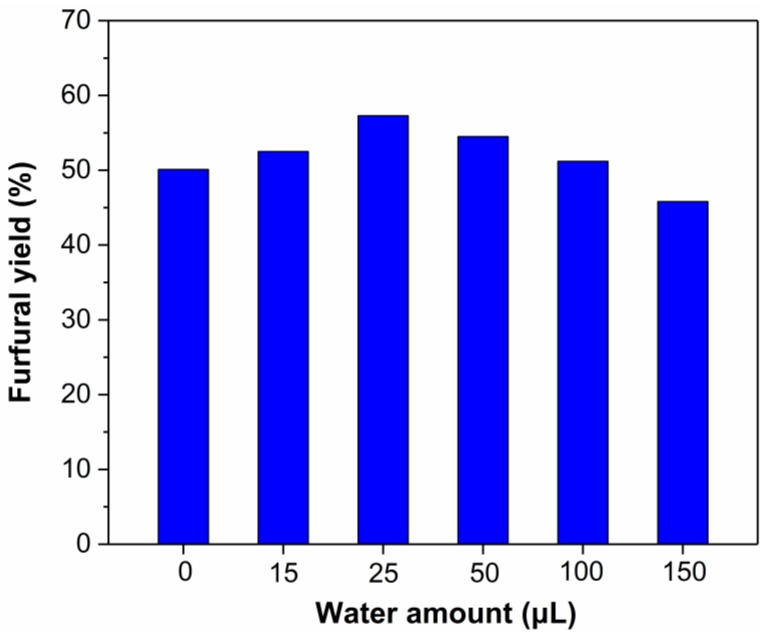
Effect of water amount on the conversion of xylan into furfural. Reaction conditions: xylan 200 mg (1.5 mmol, based on monosaccharide units); 1000 mg of EMIMBr; molar ratio of SnCl_4_:xylan = 1:10; 130 °C.

The hydrolysis of xylan to xylose requires the participation of some water (1 mol water/mol xylose unit in xylan) [19]. When there is not enough water in the reaction medium, dehydration of xylose into furfural is favored but the depolymerization of xylan will be suppressed [75]. Conversely, polymeric carbohydrates will no longer be soluble in ionic liquids and xylose dehydration will be inhibited when water content exceeds a certain level [76]. The effect of water amount on the conversion of xylan into furfural was investigated to improve the catalytic effect of the reaction system. As expected, the yield of furfural increased initially, then decreased gradually with increasing water amount (Figure 10). The furfural yield reached a maximum of 57.3% when 25 μL water was added to the reaction system. Compared with the reaction systems without additional water, an increase in the furfural yield was observed with water amounts increasing from 15 to 100 μL. When the water amount increased to 150 μL, the furfural yield was lower than that of the reaction system without water. The decreased furfural yield indicated excessive water was adverse to the production of furfural.

Binder et al. found that although CrCl_2_ and CrCl_3_ could afford furfural yields between 30–41%, the furfural yields from xylan were less than 8% in these reaction systems [22]. The highest furfural yield of 18% was obtained when adding HCl to the reaction mixture as a co-catalyst for xylan saccharification and using EMIMCl as the solvent. We attempted to use acid as a co-catalyst for xylan saccharification to improve furfural synthesis. In this study, when 50 μL 0.1mol/L HCl was added to the EMIMBr/SnCl_4_ system the furfural yield (54.6%) was almost equal to the furfural yield (54.5%) where 50 μL pure water was used. These results indicated that there was no need to employ additional Bronsted acid to promote the depolymerization of xylan, as was consistent with previous studies. Yu et al. reported that SnCl_4_ could generate Bronsted acidity in water to catalyze the hydrolysis of starch into glucose [77]. Enslow et al. demonstrated that the hydrolysis of SnCl_4_ provided sufficient Bronsted acidity to catalyze the dehydration of the ketose carbohydrates at a rate higher than the isomerization of the aldose carbohydrates [33]. Although SnCl_4_ may hydrolyze in water, leading to the formation of complex Sn species, its catalytic performance can be readily recovered by replenishing appropriate HCl.

### 2.7. Conversion of Corn Stalk into Furfural

**Figure 11:**
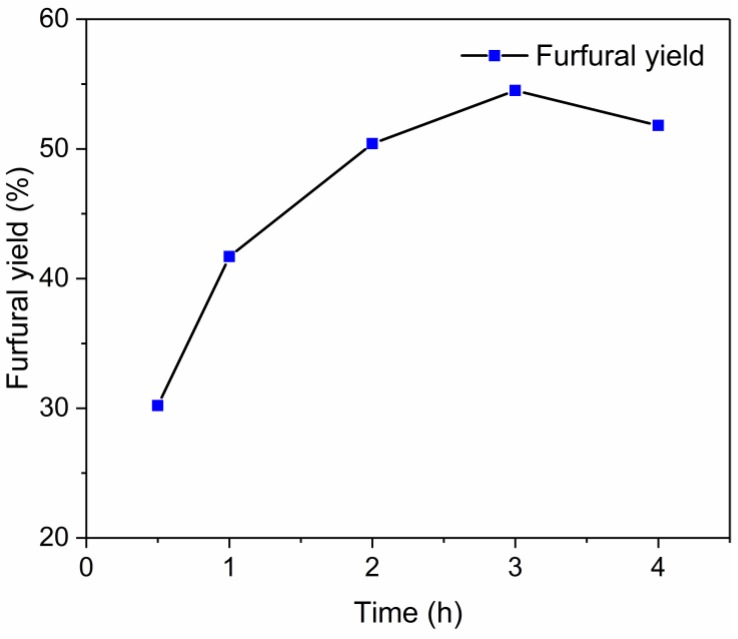
Time profile for the conversion of lignocellulosic biomass to furfural in the EMIMBr/SnCl_4_ system. Reaction conditions: corn stalk 50 mg; 1000 mg of EMIMBr; 7 μL water; 23 mg SnCl_4_; 130 °C.

The catalytic performance of the EIMIM/SnCl_4_ system was also evaluated using corn stalk as representative biomass materials as substrate. The corn stalk used in this study contained 17.9 wt% xylan, and the furfural yield was calculated based on this data. Figure 11 illustrates that a high furfural yield of 54.5% from real raw biomass was obtained at 130 °C after 3 h when a relative low substrate loading (5 wt%) was used. In addition, compared to xylose and xylan, when corn stalk was used as substrate, a longer reaction time was needed to obtain the maximal furfural yield. When the substrate loading increased to 10 wt% and 20 wt%, the furfural yields decreased to 46.4% and 34.7%, respectively, and were lower than that obtained from pure xylose or xylan. This may be due to the complexity and recalcitrance of lignocellulose [78]. 

The catalytic performance of the EMIMBr/SnCl_4_ system developed in this work was compared with other representative homogeneous reaction systems. As shown in Table 4, higher furfural yield could be obtained from the system EMIMBr/SnCl_4_ than other systems under mild conditions. Zhang et al. investigated the conversion of lignocellulosic biomass into furfural in system BMIMCl/AlCl_3_ under microwave irradiation. The furfural yields from untreated corncob, grass, and pine wood were 19.1%, 31.4%, and 33.6%, respectively, at a low substrate loading of 2.5 wt% [24]. Morais proposed a two-stage furfural production process, where wheat straw was subjected to a high-pressure CO_2_–H_2_O treatment to yield a hemicellulose hydrolysate that was then used as feed in the CO_2_-catalysed dehydration step in a multiphasic CO_2_/water/THF system with MIBK as the extracting solvent [79]. Through this process, a furfural yield of 43 mol% with a selectivity of 44 mol% was obtained with 50 bar of initial CO_2_ pressure at 180 °C, 60 min. Compared with previous reports, the EMIMBr/SnCl_4_ was more efficient for the production of furfural from raw biomass.

To the best of our knowledge, the EMIMBr/SnCl_4_ system is the most efficient reaction system for the conversion of high concentration (20 wt%) xylose, and xylan and lignocellulosic biomass. However, more work is necessary to further improve this system in order to achieve large scale application. On the one hand, the use of expensive imidazolium-based ionic liquids as reaction media results in the high cost of converting xylose into furfural. Recently, some concerns have been pointed out regarding the environmental implications and the ‘‘greenness” of processes based in ionic liquids [81,82,83]. Therefore, it is very necessary to search for alternative solvents to alleviate the disadvantages of ionic liquids, such as biomass derived ionic liquids and eutectic solvents (DESs). On the other hand, environmentally friendly and efficient extracting agents should be developed to improve the product separation and purification processes, as well as to reduce the associated environmental pollution. Besides, it is also very important to provide more evidence to confirm the proposed reaction pathway, which may promote the development of a more efficient reaction system.

## 3. Materials and Methods

### 3.1. Materials 

Furfural (99%) was purchased from Tianjin Heowns Biochem LLC (Tianjin, China). Xylose (99%), AlCl_3_·6H_2_O, and EMIMBr were purchased from Shanghai Macklin Biochemical Co., Ltd (Shanghai, China). SnCl_4_·5H_2_O, FeCl_2_·4H_2_O, FeCl_3_·6H_2_O, SnCl_2_·2H_2_O MgCl_2_·6H_2_O, CrCl_3_·6H_2_O, and THF (99%) were purchased from Tianjin Fengchuan Reagent Technologies Co., Ltd (Tianjin, China). EGDE (99%) and DMC (99%) were purchased from Tianjin Guangfu Fine Chemical Research Institute (Tianjin, China). MIBK (99%) was purchased from Adamas Reagent Co., Ltd (Shanghai, China). Toluene (99%) was purchased from Tianjin Chemical Reagent Supply and Marketing Company (Tianjin, China). BMIMBr, BMIMCl, EMIMBF_4_, and DMIMI were purchased from Shanghai Chengjie Chemical Reagent Co., Ltd. (Shanghai, China). EMIMCl was purchased from Lanzhou Institute of Chemical Physics, Chinese Academy of Sciences (Lanzhou, China). Xylan (from corncob, 95%) was purchased from Shanghai Meryer Chemical Technology Co., Ltd (Shanghai, China). Fe_2_(SO_4_)_3_ and FeSO_4_ were purchased from Tianjin Bodi Chemical Co., Ltd (Tianjin, China). HCl (36%) was purchased from Tianjin No. 5 Chemical Reagent Factory (Tianjin, China). All chemical reagents were commercially available and used without further purification.

### 3.2. Conversion of Xylose and Xylan into Furfural

Catalytic reactions were typically performed in a thick-walled glass vial (15 mL) using 10 mol% catalyst (with respect to monosaccharide), a certain amount of substrate, and 1000 mg of ionic liquid at 120–140 °C. The glass vial was sealed with a polytetrafluoroethylene plug and then heated in an oil bath, and the magnetic stirring rate was maintained at 600 rpm [42]. After the reaction, the glass vial was quenched in a cold-water bath to terminate the reaction instantly. The reaction mixture was diluted with a known mass of deionized water (for pure IL). For the biphasic system, the reaction mixture was diluted with methanol, and then the extraction phase and reaction phase were separated using a syringe and needle to evaluate the partition of furfural between the extraction phase and the reaction phase. The sample was filtered with a 0.45 μm polytetrafluoroethylene filter membrane prior to high-performance liquid chromatography (HPLC) analysis. Each experiment was performed at least three times, and the reproducibility of xylose conversion, furfural yield, and selectivity was within 3% standard deviation. The corn stalk used in this study was collected from Lianyungang, China.

All reaction products were analyzed by high performance liquid chromatography (HPLC) and quantified using calibration curves. Furfural was determined at 275 nm and 35 °C by an HPLC system equipped with a diode array detector (DAD) using a methanol/water (*v*:*v* = 70%:30%) mobile phase at a flow rate of 1.0 mL/min. Xylose was analyzed by HPLC system equipped with a refractive index detector (refractomax 521 Model) and a Shodex Sugar SH1011 analytical column (8.0 mm × 300 mm). A 5 mM H_2_SO_4_ solution was used as the mobile phase at a flow rate of 1.0 mL/min at 65 °C. The catalytic performance of the reaction system was evaluated by xylose conversion, furfural yield, and selectivity. The xylose conversion, furfural yield, and selectivity were calculated as follows:

(1) $$\mathrm{Xylose}\;\mathrm{conversion}\;(\mathrm{from}\;\mathrm{xylose})=\frac{\mathrm{moles}\;\mathrm{of}\;\mathrm{xylose}\;\mathrm{reacted}}{\mathrm{moles}\;\mathrm{of}\;\mathrm{initial}\;\mathrm{xylose}\;}\times 100\%$$

(2) $$\mathrm{Furfural}\;\mathrm{selectivity}\;(\mathrm{from}\;\mathrm{xylose})=\frac{\mathrm{moles}\;\mathrm{of}\;\mathrm{xylose}\;\mathrm{produced}}{\mathrm{moles}\;\mathrm{of}\;\mathrm{xylose}\;\mathrm{reacted}}\times 100\%$$

(3) $$\mathrm{Furfural}\;\mathrm{yield}\;(\mathrm{from}\;\mathrm{xylose})=\frac{\mathrm{moles}\;\mathrm{of}\;\mathrm{furfural}\;\mathrm{produced}}{\mathrm{moles}\;\mathrm{of}\;\mathrm{initial}\;\mathrm{xylose}}\times 100\%$$

(4) $$\mathrm{Furfural}\;\mathrm{yield}\;(\mathrm{from}\;\mathrm{xylan})=\frac{\mathrm{moles}\;\mathrm{of}\;\mathrm{furfural}\;\mathrm{produced}}{\mathrm{moles}\;\mathrm{of}\;\mathrm{initial}\;\mathrm{xylan}}\times 100\%$$

(5) $$\mathrm{Furfural}\;\mathrm{yield}\;(\mathrm{from}\;\mathrm{corn}\;\mathrm{stalk})=\frac{\mathrm{moles}\;\mathrm{of}\;\mathrm{furfural}\;\mathrm{produced}}{\mathrm{moles}\;\mathrm{of}\;\mathrm{initial}\;\mathrm{xylan}\;\mathrm{in}\;\mathrm{corn}\;\mathrm{stalk}}\times 100\%$$

## 4. Conclusions

This study investigated the catalytic performance of metal chlorides in different solvents for xylose dehydration into furfural. The EMIMBr/SnCl_4_ system was screened as the most effective reaction system, obtaining a furfural yield as high as 71.1% when the reaction was carried out at 130 °C for 1 h. In addition, the furfural yield still reached 68.8% when 10 mol% SnCl_4_ was replaced by the combined catalyst consisting of 5 mol% MgCl_2_ and 5 mol% SnCl_4_. Since the reaction was conducted at relative conditions using high xylose loading (20 wt%), the conversion efficiency of the EMIMBr/SnCl_4_ was considerably higher than other catalytic systems. Furthermore, the EMIMBr/SnCl_4_ system was also efficient to directly catalyze the conversion of xylan into furfural without the assistance of additional Bronsted acid. The addition of a certain amount of water could promote the conversion of xylan in the EMIMBr/SnCl_4_ system, obtaining a furfural yield up to 57.3%. Finally, 54.5% furfural yield from corn stalk could be obtained from the EMIMBr/SnCl_4_ reaction system.

## Figures and Tables

**Table 1:** Effect of different dosage of SnCl_4_ and MgCl_2_ on the dehydration of xylose into furfural.

Entry	SnCl_4_ (mol%)	MgCl_2_ (mol%)	Furfural Yield (%)
1	10	0	71.1
2	8	2	69.8
3	8	0	62.0
4	5	5	68.8
5	5	0	52.4
6	0	10	4.5

Reaction conditions: 200 mg xylose, 1000 mg of EMIMBr; 130 °C; 1 h.

**Table 2:** Effect of biphasic system on the conversion of xylose into furfural ^a^.

Entry	Reaction Phase	Extraction Phase	Furfural Yield (%)		
			Extraction Phase	Reaction Phase	Total
1 ^b^	water	/	/	27.3	27.3
2 ^c^	water	toluene	6.7	25.6	32.3
3 ^c^	water	DMC	7.0	19.1	26.1
4 ^c^	water	MIBK	5.9	39.8	45.7
5 ^d^	EMIMBr	THF	18.2	1.0	19.2
6 ^d^	EMIMBr	toluene	15.3	7.0	22.3
7 ^d^	EMIMBr	DMC	8.0	0.3	8.3
8 ^d^	EMIMBr	EGDE	7.2	4.6	11.8
9 ^d^	EMIMBr	MIBK	0	4.7	4.7

^a^ Reaction conditions: molar ratio of SnCl_4_:xylose = 1:10; 130 °C, 1 h. ^b^ 100 mg xylose; 500 mg water. ^c^ 100 mg xylose; 500 mg water, 2 mL organic solvent. ^d^ 100 mg xylose; 500 mg EMIMBr, 2 mL organic solvent.

**Table 3:** Comparisons of catalytic performance of the EMIMBr/SnCl_4_ system with representative catalytic systems for conversion of xylose to furfural.

Catalyst	Solvent	T/°C	t/h	Loading (wt%)	Con. (%)	Yield (%)	Sel. (%)	Ref.
CrCl_3_ + HCl	water	145	1	1		39		[65]
CrCl_3_ + HCl	water–toluene	145	2	0.5	95.8	76.3	79.6	[65]
BMIMCl–AlCl_3_	water–GVL	140	2	3	99.7	79.8	80	[59]
FeCl_3_	water–2-MTHF	140	2	3.2		71		[34]
terephthalic acid	water–toluene	190	3	3.7	91.8	70.9	77.2	[66]
SnCl_4_ + LiCl	water–DMSO	130	3	10		63		[47]
HCl	water	140	3	11.3		30		[68]
SnCl_4_	water	140	5	11.3	55	32	58	[33]
SnCl_4_	water–n-butanol	140	5	4.3	90	77	85	[33]
CO_2_–H_2_O	water–THF	180	1	1.3	82.9	69.4	83.7	[67]
SnCl_4_	EMIMBr	130	1	20	98.9	71.1	71.9	This work
SnCl_4_	EMIMBr	130	1	40		54.2		This work

**Table 4:** Comparisons of catalytic performance of the EMIMBr/SnCl_4_ system with representative catalytic systems for conversion of lignocellulosic biomass to furfural.

Substrate	Catalyst	Solvent	T/°C	t/h	Loading/wt%	Yield/%	Ref.
corn stalk	CrCl_2_ + HCl	EMIMCl	140	1		22	[22]
corncob	AlCl_3_	BMIMCl	160	0.05	2.5	19.1	[24]
grass	AlCl_3_	BMIMCl	160	2	2.5	31.4	[24]
pine wood	AlCl_3_	BMIMCl	160	2	2.5	33.6	[24]
corn stalk	BMIMCl–AlCl_3_	water–GVL	140	4	3	48.0	[59]
wheat straw	BMIMHSO_4_	BMIMHSO_4_	160	2.5	10	36.2	[80]
wheat straw	CO_2_–H_2_O	water–THF/MIBK	180	1		44	[79]
SnCl_4_	SnCl_4_	EMIMBr	130	3	5	54.5	This work
SnCl_4_	SnCl_4_	EMIMBr	130	3	10	46.4	This work

## References

[1] Lozano F.J., Lozano R. (2018). Assessing the potential sustainability benefits of agricultural residues: Biomass conversion to syngas for energy generation or to chemicals production. J. Clean. Prod..

[2] Gandarias I., García-Fernández S., Obregón I., Agirrezabal-Telleria I., Arias P.L. (2018). Production of 2-methylfuran from biomass through an integrated biorefinery approach. Fuel Process. Technol..

[3] Di Gioia M., Nardi M., Costanzo P., De Nino A., Maiuolo L., Oliverio M., Procopio A. (2018). Biorenewable deep eutectic solvent for selective and scalable conversion of furfural into cyclopentenone derivatives. Molecules.

[4] Antonetti C., Bonari E., Licursi D., Nassi o Di Nasso N., Raspolli Galletti A. (2015). Hydrothermal conversion of giant reed to furfural and levulinic acid: Optimization of the process under microwave irradiation and investigation of distinctive agronomic parameters. Molecules.

[5] Kobayashi H., Yabushita M., Komanoya T., Hara K., Fujita I., Fukuoka A. (2013). High-yielding one-pot synthesis of glucose from cellulose using simple activated carbons and trace hydrochloric acid. ACS Catal..

[6] Zhou L., Yang X., Xu J., Shi M., Wang F., Chen C., Xu J. (2015). Depolymerization of cellulose to glucose by oxidation-hydrolysis. Green Chem..

[7] Rizal N., Ibrahim M., Zakaria M., Abd-Aziz S., Yee P., Hassan M. (2018). Pre-treatment of oil palm biomass for fermentable sugars production. Molecules.

[8] Peleteiro S., Garrote G., Santos V., Parajó J.C. (2014). Furan manufacture from softwood hemicelluloses by aqueous fractionation and further reaction in a catalyzed ionic liquid: A biorefinery approach. J. Clean. Prod..

[9] Parejas A., Montes V., Hidalgo-Carrillo J., Sánchez-López E., Marinas A., Urbano F. (2017). Microemulsion and sol-gel synthesized ZrO_2_-MgO catalysts for the liquid-phase dehydration of xylose to furfural. Molecules.

[10] Alonso D.M., Bond J.Q., Dumesic J.A. (2010). Catalytic conversion of biomass to biofuels. Green Chem..

[11] Liu S., Abrahamson L.P., Scott G.M. (2012). Biorefinery: Ensuring biomass as a sustainable renewable source of chemicals, materials, and energy. Biomass Bioenergy.

[12] Werpy T.A., Holladay J.E., White J.F. (2004). Top value added chemicals from biomass: I. results of screening for potential candidates from sugars and synthesis gas. Synth. Fuels.

[13] Mariscal R., Maireles-Torres P., Ojeda M., Sádaba I., Granados M.L. (2016). Furfural: A renewable and versatile platform molecule for the synthesis of chemicals and fuels. Energy Environ. Sci..

[14] Zeitsch K.J. (2000). The Chemistry and Technology of Furfural and Its Many by-Products.

[15] Casoni A.I., Hoch P.M., Volpe M.A., Gutierrez V.S. (2018). Catalytic conversion of furfural from pyrolysis of sunflower seed hulls for producing bio-based furfuryl alcohol. J. Clean. Prod..

[16] Lv G., Chen S., Zhu H., Li M., Yang Y. (2018). Determination of the crucial functional groups in graphene oxide for vanadium oxide nanosheet fabrication and its catalytic application in 5-hydroxymethylfurfural and furfural oxidation. J. Clean. Prod..

[17] Rogowski J., Andrzejczuk M., Berlowska J., Binczarski M., Kregiel D., Kubiak A., Modelska M., Szubiakiewicz E., Stanishevsky A., Tomaszewska J. (2017). WxC-β-SiC nanocomposite catalysts used in aqueous phase hydrogenation of furfural. Molecules.

[18] Hronec M., Fulajtárova K., Liptaj T., Prónayová N., Soták T. (2015). Bio-derived fuel additives from furfural and cyclopentanone. Fuel Process. Technol..

[19] Peleteiro S., Rivas S., Alonso J.L., Santos V., Parajo J.C. (2016). Furfural production using ionic liquids: A review. Bioresour. Technol..

[20] Mamman A.S., Lee J.M., Kim Y.C., Hwang I.T., Park N.J., Hwang Y.K., Chang J.S., Hwang J.S. (2008). Furfural: Hemicellulose/xylosederived biochemical. Biofuels Bioprod. Biorefin..

[21] Agirrezabal-Telleria I., Gandarias I., Arias P.L. (2014). Heterogeneous acid-catalysts for the production of furan-derived compounds (furfural and hydroxymethylfurfural) from renewable carbohydrates: A review. Catal. Today.

[22] Binder J.B., Blank J.J., Cefali A.V., Raines R.T. (2010). Synthesis of furfural from xylose and xylan. ChemSusChem.

[23] Yang Y., Hu C.W., Abu-Omar M.M. (2012). Synthesis of furfural from xylose, xylan, and biomass using alcl3⋅6 h2o in biphasic media via xylose isomerization to xylulose. ChemSusChem.

[24] Zhang L., Yu H., Wang P., Dong H., Peng X. (2013). Conversion of xylan, d-xylose and lignocellulosic biomass into furfural using AlCl_3_ as catalyst in ionic liquid. Bioresour. Technol..

[25] Lopes M., Dussan K., Leahy J.J. (2017). Enhancing the conversion of D-xylose into furfural at low temperatures using chloride salts as co-catalysts: Catalytic combination of AlCl_3_ and formic acid. Chem. Eng. J..

[26] Ershova O., Nieminen K., Sixta H. (2017). The role of various chlorides on xylose conversion to furfural: Experiments and kinetic modeling. Chemcatchem.

[27] Gupta N.K., Fukuoka A., Nakajima K. (2017). Amorphous Nb_2_O_5_ as a selective and reusable catalyst for furfural production from xylose in biphasic water and toluene. ACS Catal..

[28] Kaiprommarat S., Kongparakul S., Reubroycharoen P., Guan G., Samart C. (2016). Highly efficient sulfonic MCM-41 catalyst for furfural production: Furan-based biofuel agent. Fuel.

[29] Nakajima K., Hirata J., Kim M., Gupta N.K., Murayama T., Yoshida A., Hiyoshi N., Fukuoka A., Ueda W. (2017). Facile formation of lactic acid from a triose sugar in water over niobium oxide with a deformed orthorhombic phase. ACS Catal..

[30] Hou Q., Zhen M., Liu L., Chen Y., Huang F., Zhang S., Li W., Ju M. (2018). Tin phosphate as a heterogeneous catalyst for efficient dehydration of glucose into 5-hydroxymethylfurfural in ionic liquid. Appl. Catal. B.

[31] Le Guenic S., Gergela D., Ceballos C., Delbecq F., Len C. (2016). Furfural Production from D-Xylose and Xylan by using stable Nafion NR50 and NaCl in a microwave-assisted biphasic reaction. Molecules.

[32] Sievers C., Musin I., Marzialetti T., Olarte M.B., Agrawal P.K., Jones C.W. (2009). Acid-catalyzed conversion of sugars and furfurals in an ionic-liquid phase. ChemSusChem.

[33] Enslow K.R., Bell A.T. (2015). SnCl_4_-catalyzed isomerization/dehydration of xylose and glucose to furanics in water. Catal. Sci. Technol..

[34] Vom S.T., Grande P.M., Leitner W., de María P.D. (2011). Iron-catalyzed furfural production in biobased biphasic systems: From pure sugars to direct use of crude xylose effluents as feedstock. Chemsuschem.

[35] Ma S., Li P., Zhu T., Chang H., Lin L. (2014). Reaction extraction of furfural from pentose solutions in a modified Scheibel column. Chem. Eng. Process..

[36] Rivas S., Gonzálezmuñoz M.J., Santos V., Parajó J.C. (2013). Production of furans from hemicellulosic saccharides in biphasic reaction systems. Holzforschung.

[37] Qing Q., Guo Q., Zhou L., Wan Y., Xu Y., Ji H., Gao X., Zhang Y. (2016). Catalytic conversion of corncob and corncob pretreatment hydrolysate to furfural in a biphasic system with addition of sodium chloride. Bioresour. Technol..

[38] Hou Q., Li W., Ju M., Liu L., Chen Y., Yang Q. (2016). One-pot synthesis of sulfonated graphene oxide for efficient conversion of fructose into HMF. RSC Adv..

[39] Ahmad T., Olsson K.T.O., Kenne L. (1995). The formation of 2-furaldehyde and formic acid from pentoses in slightly acidic deuterium oxide studied by ^1^H NMR spectroscopy. Carbohydr. Res..

[40] Siewping T., Yi G.S., Zhang Y.G. (2014). Hydroxymethylfurfural production from bioresources: Past, present and future. Green Chem..

[41] Peleteiro S., Velasco G.G., Santos V., Liñares J.C.P. (2014). Conversion of hexoses and pentoses into furans in an ionic liquid. Afinidad.

[42] Da Costa Lopes A.M., Bogel-Łukasik R. (2015). Acidic ionic liquids as sustainable approach of cellulose and lignocellulosic biomass conversion without additional catalysts. ChemSusChem.

[43] Da Costa Lopes A.M., João K.G., Bogel-Łukasik E., Roseiro L.B., Bogel-Łukasik R. (2013). Pretreatment and Fractionation of Wheat Straw Using Various Ionic Liquids. J. Agric. Food Chem..

[44] Brandt A., Ray M.J., To T.Q., Leak D.J., Murphy R.J., Welton T. (2011). Ionic liquid pretreatment of lignocellulosic biomass with ionic liquid-water mixtures. Green Chem..

[45] Hou Q., Li W., Zhen M., Liu L., Chen Y., Yang Q., Huang F., Zhang S., Ju M. (2017). An ionic liquid-organic solvent biphasic system for efficient production of 5-hydroxymethylfurfural from carbohydrates at high concentrations. RSC Adv..

[46] Yang T., Zhou Y.H., Zhu S.Z., Pan H., Huang Y.B. (2017). Insight into aluminum sulfate-catalyzed xylan conversion into furfural in a γ-valerolactone/water biphasic solvent under microwave conditions. ChemSusChem.

[47] Wang W., Li H., Ren J., Sun R., Zheng J., Sun G., Liu S. (2014). An efficient process for dehydration of xylose to furfural catalyzed by inorganic salts in water/dimethyl sulfoxide system. Chin. J. Catal..

[48] Su Y., Brown H.M., Huang X., Zhou X.D., Amonette J.E., Zhang Z.C. (2009). Single-step conversion of cellulose to 5-hydroxymethylfurfural (HMF), a versatile platform chemical. Appl. Catal. A.

[49] Chatterjee A., Hu X., Lam F.L.Y. (2018). A dual acidic hydrothermally stable MOF-composite for upgrading xylose to furfural. Appl. Catal. A.

[50] Caes B.R., Palte M.J., Raines R.T. (2013). Organocatalytic conversion of cellulose into a platform chemical. Chem. Sci..

[51] García-Sancho C., Fúnez-Núñez I., Moreno-Tost R., Santamaría-González J., Pérez-Inestrosa E., Fierro J.L.G., Maireles-Torres P. (2017). Beneficial effects of calcium chloride on glucose dehydration to 5-hydroxymethylfurfural in the presence of alumina as catalyst. Appl. Catal. B.

[52] Chang G.Y., Zhang S., Pan X. (2016). Effective conversion of biomass into bromomethylfurfural, furfural, and depolymerized lignin in lithium bromide molten salt hydrate of a biphasic system. RSC Adv..

[53] D’Anna F., Marullo S., Vitale P., Rizzo C., Meo P.L., Noto R. (2014). Ionic liquid binary mixtures: Promising reaction media for carbohydrate conversion into 5-hydroxymethylfurfural. Appl. Catal. A.

[54] Xu S., Pan D., Wu Y., Song X., Gao L., Li W., Das L., Xiao G. (2018). Efficient production of furfural from xylose and wheat straw by bifunctional chromium phosphate catalyst in biphasic systems. Fuel Process. Technol..

[55] Peleteiro S., Lopes A.M.D.C., Garrote G., Parajó J.C., Bogel-Lukasik R. (2015). Simple and efficient furfural production from xylose in media containing 1-butyl-3-methylimidazolium Hydrogen Sulfate. Ind. Eng. Chem. Res..

[56] Dibenedetto A., Aresta M., Di Bitonto L., Pastore C. (2016). Organic carbonates: Efficient extraction solvents for the synthesis of HMF in aqueous media with cerium phosphates as catalysts. ChemSusChem.

[57] Wrigstedt P., Keskiväli J., Leskelä M., Repo T. (2015). The role of salts and bronsted acids in lewis acid-catalyzed aqueous-phase glucose dehydration to 5-hydroxymethylfurfural. Chemcatchem.

[58] Gupta D., Ahmad E., Pant K.K., Saha B. (2017). Efficient utilization of potash alum as a green catalyst for production of furfural, 5-hydroxymethylfurfural and levulinic acid from mono-sugars. RSC Adv..

[59] Wang S., Zhao Y., Lin H., Chen J., Zhu L., Luo Z. (2017). Conversion of C_5_ carbohydrates into furfural catalyzed by a Lewis acidic ionic liquid in renewable γ-valerolactone. Green Chem..

[60] O’Neill R., Ahmad M.N., Vanoye L., Aiouache F. (2009). Kinetics of aqueous phase dehydration of xylose into furfural catalyzed by ZSM-5 Zeolite. Ind. Eng. Chem. Res..

[61] Antaljr M.J., Richards M.G.N. (1991). Mechanism of formation of 2-furaldehyde from d-xylose. Carbohydr. Res..

[62] Yang W., Li P., Bo D., Chang H. (2012). The optimization of formic acid hydrolysis of xylose in furfural production. Carbohydr. Res..

[63] Yemis O., Mazza G. (2011). Acid-catalyzed conversion of xylose, xylan and straw into furfural by microwave-assisted reaction. Bioresour. Technol..

[64] Dias A.S., Lima S., Pillinger M., Valente A.A. (2007). Modified versions of sulfated zirconia as catalysts for the conversion of xylose to furfural. Catal. Lett..

[65] Choudhary V., Sandler S.I., Vlachos D.G. (2012). Conversion of xylose to furfural using lewis and bronsted acid catalysts in aqueous media. ACS Catal..

[66] Hronec M., Fulajtárová K. (2018). Terephthalic acid from waste PET: An efficient and reusable catalyst for xylose conversion into furfural. Catal. Today.

[67] Morais A.R.C., Bogel-Lukasik R. (2016). Highly efficient and selective CO_2_-adjunctive dehydration of xylose to furfural in aqueous media with THF. Green Chem..

[68] Enslow K.R., Bell A.T. (2015). The role of metal halides in enhancing the dehydration of xylose to furfural. ChemCatChem.

[69] Lam E., Majid E., Leung A.C., Chong J.H., Mahmoud K.A., Luong J.H. (2011). Synthesis of Furfural from Xylose by Heterogeneous and Reusable Nafion Catalysts. ChemSusChem.

[70] Tao F.T., Song H.S., Chou L.C. (2011). Efficient process for the conversion of xylose to furfural with acidic. Can. J. Chem.

[71] Chheda J.N., Románleshkov Y., Dumesic J.A. (2007). Production of 5-hydroxymethylfurfural and furfural by dehydration of biomass-derived mono- and poly-saccharides. Green Chem..

[72] Lange J.P., Van Der Heide E., Van Buijtenen J., Price R. (2012). Furfural-a promising platform for lignocellulosic biofuels. ChemSusChem.

[73] Zhao H., Holladay J.E., Brown H., Zhang Z.C. (2007). Metal chlorides in ionic liquid solvents convert sugars to 5-hydroxymethylfurfural. Science.

[74] Zhang Z., Zhao Z.K. (2010). Microwave-assisted conversion of lignocellulosic biomass into furans in ionic liquid. Bioresour. Technol..

[75] Sen S.M., Binder J.B., Raines R.T., Maravelias C.T. (2012). Conversion of biomass to sugars via ionic liquid hydrolysis: Process synthesis and economic evaluation. Biofuels, Bioprod. Biorefin..

[76] Swatloski R.P. (2002). Dissolution of cellulose with ionic liquids. J. Am. Chem. Soc..

[77] Yu I.K.M., Tsang D.C.W., Yip A.C.K., Chen S.S., Ok Y.S., Poon C.S. (2016). Valorization of food waste into hydroxymethylfurfural: Dual role of metal ions in successive conversion steps. Bioresour. Technol..

[78] Zhang T., Li W., Xu Z., Liu Q., Ma Q., Jameel H., Chang H.M., Ma L. (2016). Catalytic conversion of xylose and corn stalk into furfural over carbon solid acid catalyst in γ-valerolactone. Bioresour. Technol..

[79] Morais A.R.C., Matuchaki M.D.D.J., Andreaus J., Bogel-Lukasik R. (2016). A green and efficient approach to selective conversion of xylose and biomass hemicellulose into furfural in aqueous media using high-pressure CO_2_ as a sustainable catalyst. Green Chem..

[80] Carvalho A.V., da Costa Lopes A.M., Bogel-Łukasik R. (2015). Relevance of the acidic 1-butyl-3-methylimidazolium hydrogen sulphate ionic liquid in the selective catalysis of the biomass hemicellulose fraction. RSC Adv..

[81] Bado-Nilles A., Diallo A.-O., Marlair G., Pandard P., Chabot L., Geffard A., Len C., Porcher J.-M., Sanchez W. (2015). Coupling of OECD standardized test and immunomarkers to select the most environmentally benign ionic liquids option-Towards an innovative “safety by design” approach. J. Hazard. Mater..

[82] Peric B., Sierra J., Martí E., Cruañas R., Garau M.A., Arning J., Bottin-Weber U., Stolte S. (2013). (Eco)toxicity and biodegradability of selected protic and aprotic ionic liquids. J. Hazard. Mater..

[83] Diallo A.-O., Fayet G., Len C., Marlair G. (2012). Evaluation of Heats of Combustion of Ionic Liquids through Use of Existing and Purpose-Built Models. Ind. Eng. Chem. Res..

